# The Preparation and Performance of Epoxy/Acetylene Carbon Black Wave-Absorbing Foam

**DOI:** 10.3390/polym16081074

**Published:** 2024-04-12

**Authors:** Xiaoli Liu, Hao Huang, Haijun Lu

**Affiliations:** 1 Composite Technology Center, AVIC Beijing Aeronautical Manufacturing Technology Research Institute, Beijing 101300, China; lxl_nanliu@163.com (X.L.);; 2 National Key Laboratory of Advanced Composites, AECC Beijing Institute of Aeronautical Materials, Beijing 100095, China

**Keywords:** epoxy, acetylene carbon black, wave absorption, foam

## Abstract

The epoxy foam material filled with an absorbing agent effectively absorbs electromagnetic waves. In this study, epoxy resin was used as the matrix, and acetylene carbon black was used as the magnetic absorbing agent to prepare an absorbing foam material (epoxy/CB). The microstructure of acetylene carbon black (CB) and its distribution in epoxy resin, as well as the effects of pre-polymerization time and CB content on the foam structure, were systematically characterized. Additionally, two dispersion methods, the hot-melt in situ stirring dispersion method and the three-roll milling dispersion method, were studied for their effects on the foaming process and absorbing properties of epoxy/CB. The results showed that with the prolongation of pre-polymerization time, the pore size decreased from 1.02 mm to 0.4 mm, leading to a more uniform pore distribution. Compared to the hot-melt in situ stirring dispersion method, the three-roll milling dispersion method effectively improved the dispersion of CB in epoxy resin, reducing the aggregate size from 300–400 nm to 70–80 nm. The pore diameter also decreased from 0.453 mm to 0.311 mm, improving the uniformity of particle size distribution. However, the absorbing material prepared with the three-roll milling dispersion method exhibited unsatisfactory absorption performance, with values close to 0 dB at mid-low frequencies and around −1 dB at high frequencies. In contrast, the absorbing material prepared with the hot-melt in situ stirring dispersion method showed better absorption performance at high frequencies, reaching around −9 dB.

## 1. Introduction

Thermosetting epoxy foam materials are commonly used in the manufacturing of lightweight, high-strength sandwich structure composite components due to their advantages of low density, high strength, damage tolerance, good heat resistance, and low moisture absorption [1,2,3,4,5,6,7,8]. Epoxy foam was first developed by the US Company Shell in the late 1940s, but it was not until the 1970s that it began to be applied. Its earliest applications were in the aviation industry, specifically in the manufacturing of the components of aircraft.

The preparation methods of epoxy foam plastics mainly include chemical foaming [9,10,11,12], physical foaming [13,14], and hollow microsphere methods [15,16,17]. The former two methods are suitable for manufacturing low- and medium-density foam plastics, while the latter method is mostly used for high-density foam plastics. The physical foaming method utilizes the heat released from the resin curing reaction to sublime or evaporate low-boiling point liquids into gas, dispersing them in the resin for foaming. The mixture is then poured into molds or sprayed to produce epoxy foam plastic products or coatings.

The preparation methods of chemical foaming mainly consist of two categories: one-step and two-step methods. The one-step method involves direct foaming and curing in the resin matrix, while the two-step method consists of pre-polymerization and foaming–curing processes. In the one-step method, due to the low resin viscosity and weak cell wall strength in the initial stages of the reaction, the formed cell structure is unstable, prone to rupture, collapse, or the creation of large voids. Additionally, the large amount of heat generated by the exothermic reaction is difficult to dissipate, leading to the risk of explosion, scorching, and other issues. These problems can be effectively addressed by the two-step method. During the pre-polymerization process, the resin system undergoes chain extension, branching, and partial cross-linking to achieve a certain degree of curing and viscosity, enough to support the bubbles generated during the foaming–curing process. The pre-polymerization reaction helps the system to dissipate some of the reaction heat, reducing the concentration of heat during the foaming–curing process, thus preventing scorching and ensuring a more stable and uniform cell structure [14,18,19,20].

Structural absorbing foam not only has excellent electromagnetic wave absorption properties but also features high strength, lightweight, and sound absorption and shock mitigation capabilities. Owing to its strong designability and integrated multifunctionality, it is suitable for the manufacturing and processing of complex-shaped and full-height sandwich components, attracting increasing attention. Park et al. [21] designed and fabricated a sandwich-type radar-absorbing structure (RAS) for X-band frequency. By incorporating conductive fillers such as carbon black and MWNT into composite panels and polyurethane foam cores, the absorption capacity of RAS was effectively enhanced. Additionally, the sandwich structure improved the mechanical strength of the RAS. Gao et al. [22] prepared asymmetrically conductive epoxy/functionalized reduced graphene oxide/nickel chain microcellular foam (a-EP/f-RGO/Ni chain microcellular foam) using a hot compression method, followed by foaming using supercritical carbon dioxide (scCO_2_). The a-EP/f-RGO/Ni chain microcellular foam with a f-RGO content of 5 vol% and a nickel chain content of 5 vol% exhibited enhanced conductivity (10^−1^ S/m). Compared to the homogenous conductive structure EP/f-RGO/Ni chain (h-EP/f-RGO/Ni chain) microcellular foam with the same filler content, it showed higher S/m in the X-band and superior EMI shielding effectiveness (40.82 dB). Lei Yu et al. [23] prepared cement-based foam composite materials with different densities, MWCNT content, and various layer combinations. The optimal electromagnetic wave (EMW) absorption performance of the single-layer composite material demonstrated an effective absorption bandwidth (EAB) of 9.76 GHz and a minimum reflection loss (RL_min_) of −13.6 dB. In comparison to the single-layer samples, the double-layer samples exhibited a wider EAB of 12.7 GHz, with the RL_min_ being −32.9 dB at the same thickness. Xu et al. [24] utilized Sapiumse biferum kernel oil-based polyurethane foam (BPUF) as a porous matrix and Fe_3_O_4_ as an absorber to prepare a novel renewable microwave-absorbing foam. The results showed that the mBPUF exhibited a porous multilayer structure, providing a scaffold and matching layer for Fe_3_O_4_ nanoparticles. The effective reflection loss (RL ≤ −10 dB) frequency range for the mBPUF was from 4.16 GHz to 18 GHz. While some progress has been made in the research of structural absorbing foam, the focus has largely been on low-temperature-resistant PU foam systems. There is relatively less research on high-temperature-resistant structural absorbing foam, which cannot meet the corresponding requirements for temperature resistance, absorption, and load bearing. Therefore, it is necessary to research and develop high-temperature-resistant structural absorbing foam with good absorption performance and load-bearing capacity to meet the increasing demands for high absorption, load bearing, and temperature resistance.

Epoxy structural foam exhibits excellent heat resistance and mechanical properties, meeting the requirements for high-temperature resistance and load bearing of wave-absorbing foam. High-temperature-resistant structural absorbing foam involves adding absorbents into the resin system, which can be foamed through a reaction. The shape, size, and interface interaction between the absorbent and the polymer will affect its dispersion in the matrix [25,26,27,28,29,30]. The method of dispersing the absorbent and its distribution in the foam will also impact the cell structure and absorbing performance [31,32,33,34]. Therefore, in this work, a wave-absorbing foam material composed of epoxy resin and acetylene carbon black (CB) was first prepared using in situ mechanical stirring and the three-roll milling method. The particle morphologies in the foam matrix of the two preparation methods were compared. The effects of pre-polymerization time and CB content on the morphology and absorption properties, compression strength, and modulus of foam were analyzed.

## 2. Materials and Methods

### 2.1. Materials

**Figure 1:**
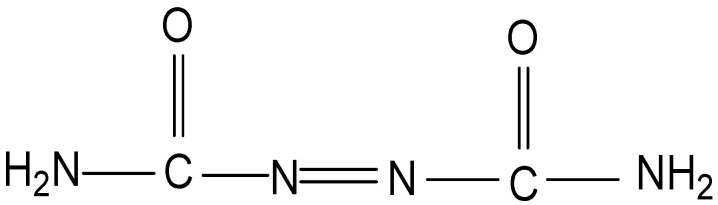
Molecular structure of azodicarbonamide.

Bisphenol A epoxy resin E51 (epoxide equivalent of 190–210 g/eqv.; viscosity of 2500 m∙Pa∙s at 40 °C) was purchased from Nantong Xingchen Synthetic Material Co., Ltd. (Nantong, China). 4,4′-Diaminodiphenylsulfone (DDS) was purchased from Hansoh High New Material (Tianjin) Co., Ltd. (Tianjin, China), and acetylene carbon black (CB) was sourced from Zhangjiakou Xiahuayuan Battery Factory (Zhangjiakou, China). The foaming agent azodicarbonamide (AC), an industrial product with a decomposition temperature of 215 °C and a gas yield of 210 mL/g, was purchased from Beijing Letai Chemical Co., Ltd. (Beijing, China), and all materials were used in the manner in which they were received. The molecular structure of the foaming agent is shown in Figure 1.

### 2.2. Preparation

Hot-melt in situ stirring method: CB was gradually added to the E51 resin by stirring the paddle until the CB was completely added. When the mixture was stirred evenly, the curing agent DDS (40% of the resin content) was added and stirred.

Three-roll milling dispersion method: E51 and DDS were mixed in a mass ratio of 100:40, and then CB was added. The mixture was further rolled to disperse the black carbon evenly using a three-roll grinder with a roll gap of 0.5–0.8 μm and a roll speed of 250–300 rpm to obtain the epoxy/CB wave-absorbing resin.

Foaming process: Resin was heated to 120–140 °C to pre-polymerize for a certain time. After that, it was cooled to 100–110 °C, and the foaming agent AC (5 wt% of the resin content) was added and mixed thoroughly to obtain the pre-foamed material. Finally, the pre-foamed material was placed in a compression mold and foamed at 180 °C for 2–3 h to obtain the absorbing foam.

### 2.3. Characterization and Measurements

Electromagnetic parameters of CB: A mixture of 10 wt% acetylene carbon black and paraffin was uniformly prepared to create a circular sample with an inner diameter of 3.04 mm, an outer diameter of 7.0 mm, and a length of 2 mm. The test was conducted using a vector network analyzer within a frequency range from 2 to 18 GHz.

The reflectivity of wave-absorbing foam: The reflectivity of the foam was tested within the frequency range from 2 to 18 GHz using an HP 8722ES vector network analyzer (Agilent Technologies Inc., Santa Clara, CA, USA). The foam sample size was 200 mm × 200 mm × 10 mm.

Scanning electron microscopy (SEM) observation: The morphology of the samples was observed with a Camscan 3100 electron microscope from the Aylesbury, UK with an acceleration voltage of 20 kV. The samples were cryogenically fractured and then sputtered with gold before observation. To calculate the average pore diameter, image-processing (Matlab R2023a For Windows) software was utilized.

Compression strength and modulus of the samples: Compression stress–strain curves were obtained using a Shimadzu AG-I universal testing machine (Tokyo, Japan) at a compression speed of 3 mm/min following ASTM D 1621-A standards [35]. The samples had dimensions of 50 mm × 50 mm × 26 mm, and five samples were tested in each group to obtain the average values.

The viscosity of the mixture was measured with a Techcomp SNB-2E digital rotary viscometer (Hefei, China).

The foam density of the cylindrical epoxy foams was quantified on cut samples and calculated as follows:

(1) $$\rho_{f}=\frac{m}{V}=\frac{4m}{\pi d^{2}h}$$

where *m* is the mass of the epoxy foam sample. To measure the skeletal density *ρ*, of the cured epoxy foam, ~0.5 g ground powder of epoxy foams was subjected to helium displacement pycnometry (AccuPyc l1 1350, Micrometrics, Aachen, Germany).

## 3. Results and Discussion

### 3.1. Absorbent Characteristics

**Figure 2:**
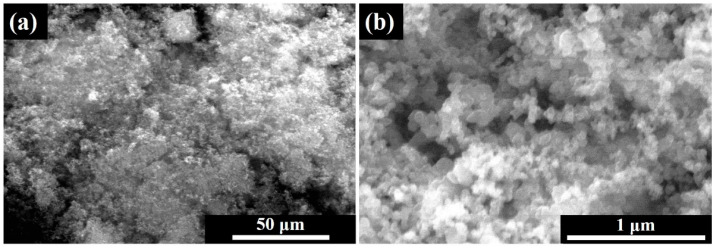
SEM images of CB: (**a**) low magnification; (**b**) high magnification.

The characteristics of the absorbent mainly include its electromagnetic parameters, particle size, shape, and aggregation state, all of which can impact the material’s absorbing properties. The SEM image of the CB is shown in Figure 2, which demonstrates that the CB is loosely aggregated, and each aggregation is composed of individual primary particles (approximately 50 nm in size). The particle size of CB is in the nanometer range, possessing a high surface energy. During the melt dispersion process, it is prone to aggregation, leading to the formation of a network structure within the filler.

**Figure 3:**
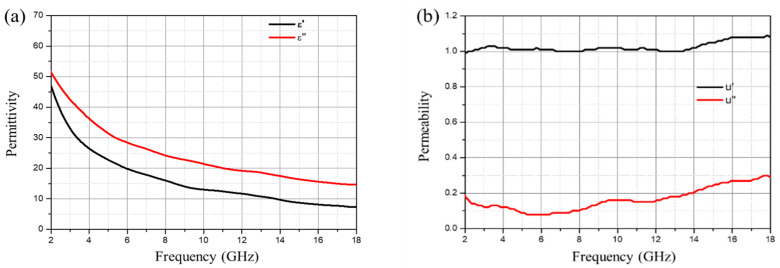
(**a**) Permittivity and (**b**) magnetic permeability of CB (10 wt%).

Figure 3 depicts the dielectric constant and magnetic permeability of 10 wt% CB. The real and imaginary parts of the permittivity are 46 and 55 at 2 GHz, respectively. As the frequency increases, they decrease linearly, reaching 8 and 15 at 18 GHz. The real and imaginary parts of the magnetic permeability are 1 and 0, respectively, showing minimal variation across the entire frequency range. Both the real and imaginary parts of the permittivity decrease with increasing frequency, indicating that CB exhibits good wave-absorbing properties at high frequencies. The loss of CB is primarily attributed to dielectric loss and occurs in dielectric loss wave-absorbing materials. As the frequency increases, the permittivity gradually decreases, which is advantageous for widening the bandwidth. When the CB content is low, the particles are uniformly and independently dispersed in the matrix with a large distance between adjacent particles, which can hardly form a conductive network, resulting in less contribution to electromagnetic wave absorption. With an increase in the CB content, CB particles may come into contact or be sufficiently close to form a continuous conductive path or network through tunneling effects or electronic transitions, leading to an improvement in electromagnetic wave absorption. Additionally, due to the small size and large specific surface area of CB particles containing a high proportion of surface atoms and a large number of dangling bonds, interface polarization and multiple scattering also become important factors in inducing wave-absorbing loss [36].

### 3.2. Distribution State of CB in Epoxy Foam

**Figure 4:**
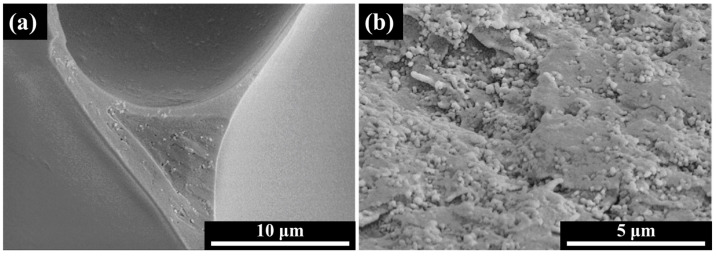
Distribution of CB in epoxy foam: (**a**) low magnification; (**b**) high magnification.

The distribution state of the absorber in foam materials is an important factor affecting its wave-absorbing performance [37]. The distribution state of the absorber in the foam is mainly determined by the absorber’s shape characteristics and the dynamic factors of pore generation and growth during the foaming process. The distribution of CB in the epoxy foam is shown in Figure 4. It can be observed from the figure that the distribution of CB in the epoxy foam is non-uniform, and the CB particles mainly aggregate at the nodes of the foam’s network structure. This situation is primarily caused by the Gibbs–Marangoni effect, exudation, and gravitational effects, which induce the flow of the liquid film, leading to the relative displacement of CB particles along the liquid film. Simultaneously, due to the re-agglomeration of CB and the capillary action of the liquid, CB particles are bonded, ultimately causing the aggregation of CB toward the exudation region. As a result, the majority of the CB is concentrated at the nodes of the network structure in the epoxy/CB foam, making it difficult for CB particles to form interconnected conductive paths in this system.

**Figure 5:**
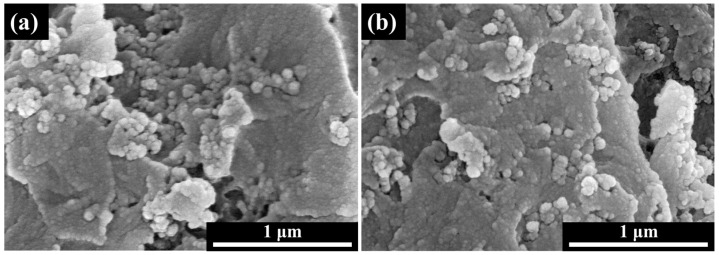
Cross-sectional SEM images of epoxy/CB resin prepared using different dispersion methods: (**a**) in situ mechanical stirring; (**b**) three-roll milling.

The interface interaction with polymers, size, and the morphology of the absorber will affect its dispersion in the polymer matrix. Moreover, the response to the same dispersion method varies depending on the shape of the absorber. Figure 5 shows the cross-sectional SEM images of the epoxy/CB composites prepared with in situ mechanical stirring and three-roll milling. It can be observed that severe CB aggregation occurs during mechanical stirring, with an aggregate size of approximately 300–400 nm, resulting in poor dispersion. However, after three-roll milling, the large aggregates are broken down, and the aggregate size reduces to approximately 70–80 nm, indicating improved dispersion of the CB. During mechanical stirring, the shear force is relatively small, making it difficult to break up the CB aggregates, leading to severe aggregation. In contrast, strong shear forces are generated between the rolls during three-roll milling, which effectively disperses the CB aggregates, resulting in good dispersion. CB is a high-surface-energy material with weak adsorption to organic substances and is prone to particle re-agglomeration, leading to poor dispersion in the epoxy matrix and weakening the effectiveness of CB. However, under the action of strong shear forces, the CB aggregates can be broken apart, disrupting their network structure and achieving good dispersion.

**Figure 6:**
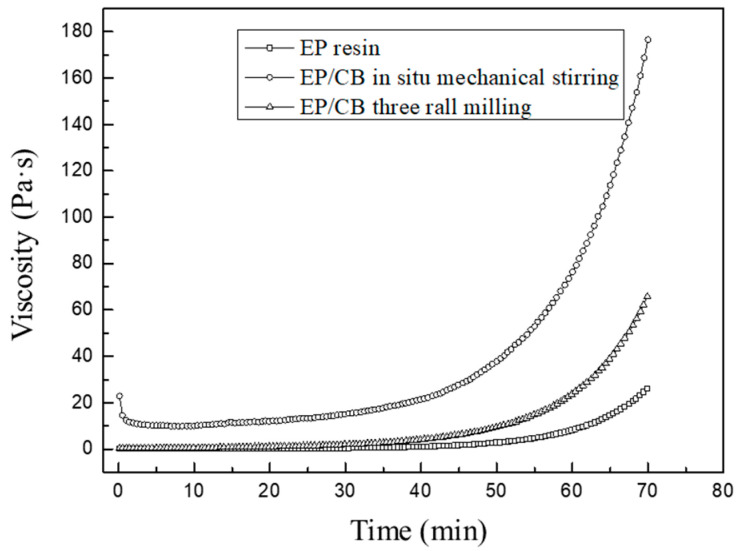
Influence of dispersion process on the viscosity characteristics of epoxy/CB resin (constant temperature at 140 °C).

Viscosity has a significant impact on the formation of foam materials, directly determining the foam growth process, and higher viscosity will reduce the foam growth rate. The addition of an absorber will inevitably affect the system viscosity, thus affecting the final foam structure. The effect of the dispersion process on the constant temperature viscosity characteristics of the epoxy/CB resin is shown in Figure 6. As shown in the figure, with mechanical stirring, the initial viscosity is about 19 Pa·s, and after mechanical three-roll milling, the viscosity decreases to 1.6 Pa·s. After 50 min of pre-polymerization, the viscosity of the blank resin is 6 Pa·s. When CB is dispersed using mechanical stirring, the viscosity quickly rises to 35 Pa·s, and after dispersion using three-roll milling, the viscosity is 9.5 Pa·s. It can be seen that regardless of the dispersing method used, the addition of CB causes an increase in system viscosity. Compared to in situ mechanical stirring, three-roll milling has a smaller impact on viscosity and therefore has a lesser impact on the foaming process. This is because three-roll milling generates strong shear forces that help to disperse the CB and break the network structure of CB aggregates. The addition of filler particles generally increases the system viscosity, leading to processing difficulties. Compared to in situ mechanical stirring, three-roll milling has a smaller impact on viscosity, which is beneficial for processing performance.

**Figure 7:**
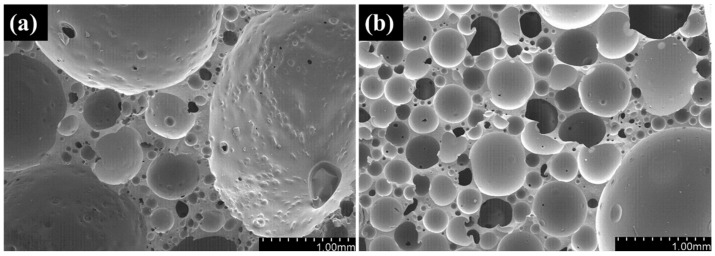
Effect of dispersion methods on foam morphology: (**a**) in situ mechanical stirring; (**b**) three-roll milling.

**Figure 8:**
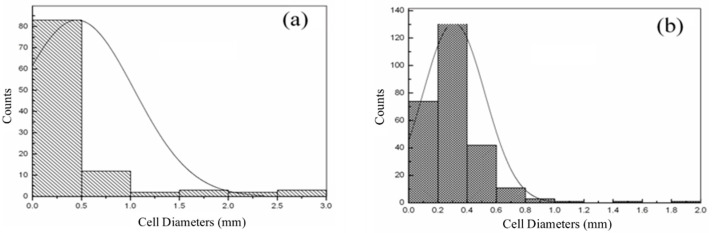
Impact of dispersion methods on the pore size distribution of epoxy/CB foam: (**a**) in situ mechanical stirring; (**b**) three-roll milling.

The effect of the dispersion methods on the pore structure of epoxy/CB foam is shown in Figure 7, and the pore size distribution is shown in Figure 8. Compared to mechanical stirring, after three-roll milling, the pore diameter decreases from 0.453 mm to 0.311 mm, and the uniformity of pore size distribution improves. This is because the addition of CB acts as a nucleating agent. For three-roll milling dispersion, these uniformly dispersed CB particles act as nucleation points. During the foaming process, a large number of nucleation points start to foam simultaneously; however, only a small amount of gas is available for foam growth, leading to a reduction in pore size. The number of nucleation points is only related to the quantity and particle size of the nucleating agent. When using in situ mechanical stirring, more CB particles exist in the form of aggregates, resulting in larger but fewer nucleation points. Hence, the nucleation density decreases, and the pore size increases.

**Figure 9:**
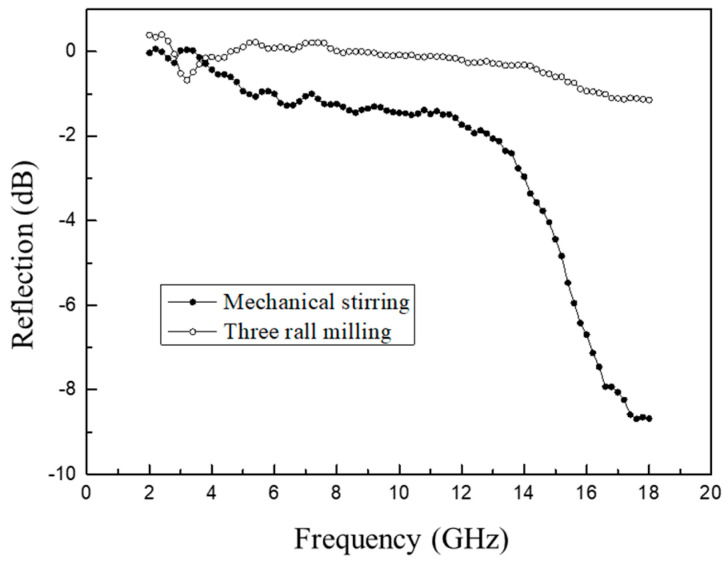
Reflectivity of foams prepared by three-roll milling and in situ mechanical stirring.

The impact of the dispersion process on the absorption performance of the CB/epoxy structural absorption foam is illustrated in Figure 9. As depicted in the graph, the foam prepared by three-roll milling exhibits a reflectivity close to 0 in the 2–16 GHz range and a reflectivity of around −1 dB in the 16–18 GHz range. Conversely, the foam produced by mechanical stirring shows a reflectivity of approximately −2 dB in the 4–14 GHz range, which rapidly decreases with increasing frequency after 14 GHz, reaching a reflectivity of −9 dB at 18 GHz. The analysis above reveals that the foam prepared by three-roll milling has well-dispersed CB; lower viscosity; and a finer, more uniform pore structure, which may be advantageous for mechanical properties, but the absorption performance is not ideal. This is related to the distribution state of the CB in the epoxy structural absorption foam. The CB in the epoxy foam is unevenly distributed, mainly concentrated at the triangular nodes, insufficient to form a chain-like conductive path in contact, and the tunnel effect is primarily present. Three-roll milling generates strong shear, which can lead to the over-dispersion of the CB, disrupting the network structure of CB, and thus resulting in deteriorated absorption performance. In contrast, melt mechanical stirring, static interactions, and the lack of shear force mean that CB particles are mainly distributed on the polymer surface, making it easier to form a conductive network at lower concentrations.

### 3.3. Influence of Pre-Polymerization Time on Pore Structure and Properties

The addition of the absorber leads to an increase in viscosity, which will inevitably affect the subsequent pre-polymerization and foaming processes. Therefore, this section examines the influence of pre-polymerization on the pore structure of the epoxy wave-absorbing foam, providing a reference for the preparation of foam materials with uniform pores and good performance.

**Figure 10:**
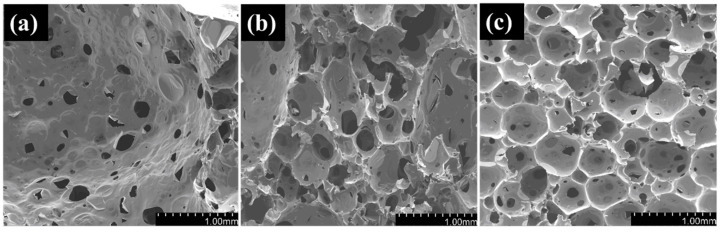
Effect of pre-polymerization time on the pore structure of CB/epoxy foam (2 wt% CB content): (**a**) 0 min; (**b**) 20 min; (**c**) 40 min.

**Figure 11:**
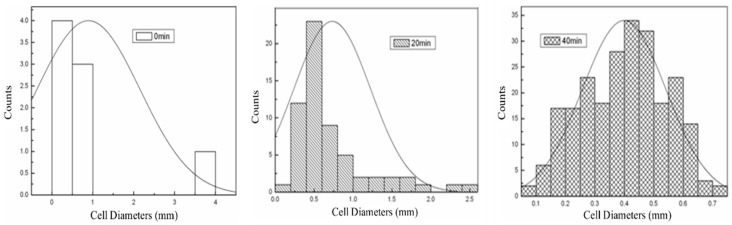
Effect of pre-polymerization time on the pore size distribution of CB/epoxy foam.

The effect of pre-polymerization time on the pore structure and size distribution of the epoxy/CB foam is shown in Figure 10 and Figure 11. The pores still exhibit a closed-cell spherical structure, indicating that the addition of CB does not alter the foam’s pore structure. With the extension of pre-polymerization time, the pore diameter decreases from 1.02 mm to 0.4 mm, and the size distribution follows a typical normal distribution. As the pre-polymerization time increases, the peak shifts toward a smaller size, and the distribution becomes more uniform. This is because, with the extension of pre-polymerization time, the increase in system viscosity due to resin cross-linking leads to enhanced foam growth stability and improved uniformity, as it matches the gelation characteristics of the resin with the decomposition rate of the foaming agent. A longer pre-polymerization time results in a reduction in the pore diameter, which is advantageous for the mechanical properties of the foam. Additionally, the increase in resin viscosity with longer pre-polymerization time reduces the occurrence of foam collapse, leading to the improved processing properties of the foam. However, when the pre-polymerization time exceeds 40 min, the viscosity becomes excessively high, causing difficulty in the system flow and making it impossible to fabricate the foam.

### 3.4. Impact of CB Content on Pore Structure and Performance of Foam

**Figure 12:**
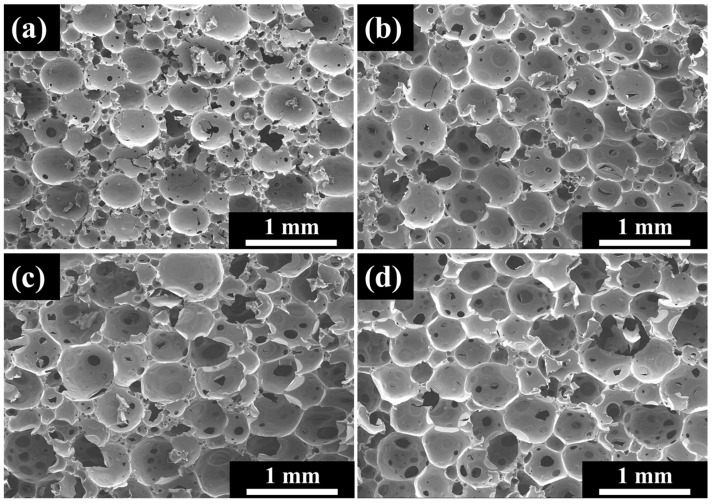
Influence of CB content on pore structure (pre-polymerization time of 40 min): (**a**) 0 wt% CB content; (**b**) 1 wt% CB content; (**c**) 2 wt% CB content; (**d**) 3 wt% CB content.

Figure 12 illustrates the influence of the CB content on foam morphology. Mechanical stirring was used to disperse the CB, which had a significant impact on the system viscosity, thus limiting the amount of CB added. When the CB content exceeds 3 wt%, it becomes difficult to achieve the designated pre-polymerization degree (pre-polymerization for 40 min). It can be observed from the graph that with the increase in the CB content, the pore diameter slightly decreases, but the overall change is not substantial, remaining around 0.4 mm. The addition of the CB serves as a nucleating agent. The nucleating effect of the nucleating agent is much greater than that of the free space of the molecule. However, the number of nucleation points is only related to the quantity and particle size of the nucleating agent. Since mechanical stirring was employed, the aggregation of CB was relatively low, mostly existing in the form of aggregates. The number of aggregates is far smaller than that of free space points, hence resulting in larger pore sizes. With an increase in the amount of CB, the change is minimal.

**Figure 13:**
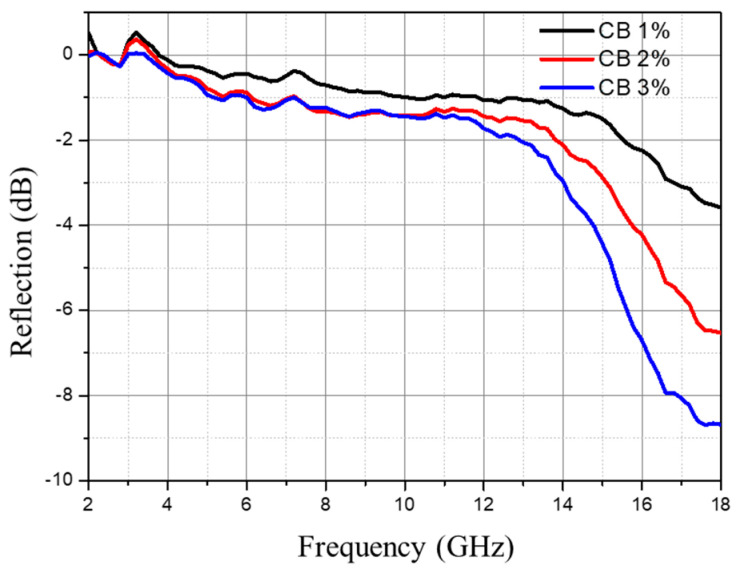
Influence of CB content on foam wave-absorbing performance (10 mm thickness).

**Figure 14:**
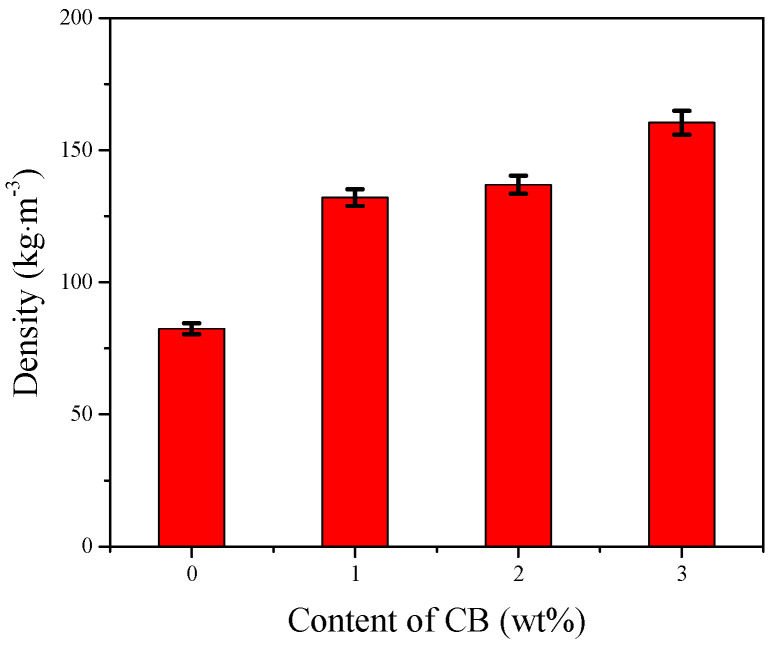
Influence of CB content on foam density.

The influence of the CB content on the foam’s wave-absorbing performance is depicted in Figure 13, while the corresponding foam density is illustrated in Figure 14. With an increase in the CB content, the microwave absorption performance gradually enhances, primarily demonstrated by a decrease in reflectivity at the same frequency, a shift in the peak toward the lower frequency direction, and a gradual widening of the effective bandwidth. As the CB content increases, the foam density also increases. This is due to the increase in system viscosity resulting from the higher CB content, which limits foam expansion and subsequently raises foam density. Additionally, the increased system viscosity and reduced flowability also contribute to higher foam density. The increase in the CB content and the resulting higher density lead to a higher concentration of CB particles per unit volume. This results in a greater number of CB particles available for electromagnetic wave absorption, contributing to an increase in the dielectric constant and loss tangent, which is beneficial for electromagnetic wave absorption. Furthermore, the higher concentration of CB particles and the increased surface area aid in reflecting and attenuating electromagnetic waves, promoting the formation of a conductive network and consequently enhancing the wave-absorbing performance.

Table 1 shows the effect of the CB content on the compression properties of the epoxy wave-absorbing foam. Compared to the blank foam, the addition of CB increases the foam wall thickness, leading to improved compression strength and modulus. This is because the CB particles are primarily distributed at the cell junctures of the foam material, increasing the foam wall thickness and enhancing its resistance to deformation, thereby increasing strength and modulus. Additionally, the foam density nearly doubles, which is beneficial for the improvement in compression strength and modulus. However, with an increase in the CB content, the compression strength and modulus exhibit a trend of initially increasing and then decreasing. This is due to the mechanical dispersion of the CB used to enhance the foam’s microwave absorption performance in this study. The irregularly shaped and poorly mechanically stable CB particles, existing mostly in the form of aggregates, exhibit weak mechanical strength and poor interface interaction with the polymer, leading to a reduction in foam strength. Moreover, the presence of aggregates in the resin system can lead to stress concentration, adversely affecting the foam’s compression properties. As the CB content increases, dispersion becomes more difficult, resulting in larger and more aggregates, which in turn harm compression strength and modulus. Therefore, the compression modulus shows a maximum value at 2 wt% CB.

### 3.5. Effect of Foam Thickness on Electrical Performance

**Figure 15:**
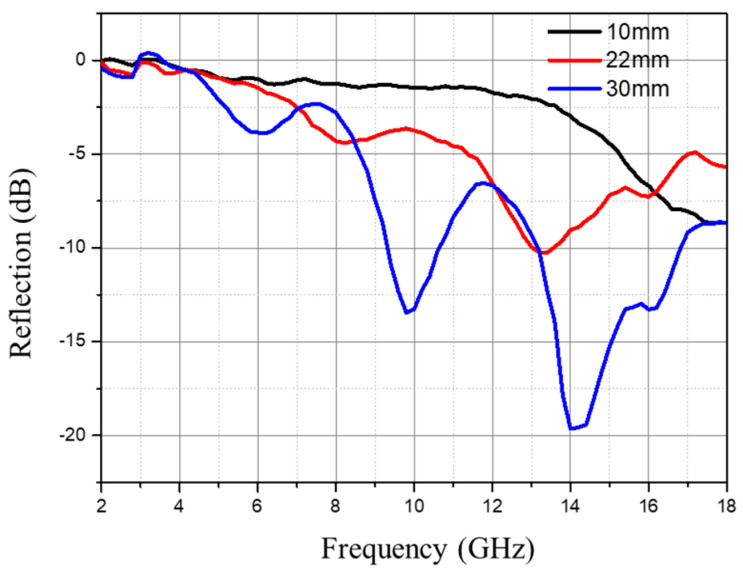
Influence of foam thickness on electrical performance (3 wt% CB content).

The influence of foam thickness on the wave-absorbing performance is shown in Figure 15. The results indicate that with an increase in foam thickness for each system, the peak reflectivity at the corresponding frequency gradually decreases and shifts toward lower frequencies, while multiple reflectivity peaks appear. In the case of the CB system, a 30 mm foam thickness results in three reflectivity peaks, measured at (5 GHz, −4 dB), (10 GHz, −14 dB), and (14 GHz, −20 dB), with a wide absorption band observed between 9 and 11 GHz and between 13 and 18 GHz, where the reflectivity is below −8 dB.

The thickness significantly influences the wave-absorbing performance of the foam. Within our testing range, with the increase in foam thickness, the absorption peak strengthens, and the absorption band widens. An increased foam thickness results in multiple reflections and the scattering of the electromagnetic waves within the foam, which increases the propagation path of the waves within the material and thus has a certain impact on wave absorption. The continual increase in thickness alters the wave impedance of the material and also changes the reflection conditions of the incident waves on the material’s surface, thus affecting the material’s absorption performance.

When the thickness of the absorbing foam equals one-fourth of the wavelength, the phase of the reflected wave at the material’s surface is opposite to that of the incident wave, leading to interference and cancellation, which maximally confines the total reflected wave inside the foam. The energy of the electromagnetic wave is dissipated by the absorbent. At this point, the thickness of the material matches the wavelength, and thus, the thicker the foam, the longer the wavelength it can match, resulting in a lower corresponding absorption frequency. This phenomenon can also be explained by the following equation [38]:

(2) $$f_{m}=\frac{c}{2\pi \mu \prime \prime d}$$

where *c* is the speed of light, and *d* is the sample thickness. Equation (1) indicates that, with an increase in sample thickness, the matching frequency shifts toward lower frequencies. Additionally, as the absorber content or thickness increases, the absorption band also shifts toward lower frequencies.

## 4. Conclusions

The present study investigated the preparation of high-temperature-resistant epoxy-absorbing foam materials with added absorber acetylene carbon black using hot-melt in situ stirring dispersion and three-roll grinding dispersion methods, and the following conclusions were drawn:

(1) CB has good absorption performance in the high-frequency range, and a lower CB content is advantageous in reducing electromagnetic wave loss. Moreover, when the CB content exceeds 3 wt%, pre-polymerization becomes difficult, making it challenging to achieve the desired degree of pre-polymerization.
(2) Mechanical stirring results in the severe aggregation of CB, with aggregate sizes of about 300–400 nm, leading to poor dispersion. However, after three-roll grinding, the large aggregates are broken down, resulting in aggregate sizes of approximately 70–80 nm, thereby improving the dispersion of CB.
(3) Although three-roll grinding dispersion results in good dispersion of CB, lower viscosity, and a fine and uniform pore structure, it may be advantageous for mechanical properties. However, the absorption performance is not ideal, as the foam prepared using three-roll grinding has nearly 0 reflectivity in the 2–16 GHz range and a reflectivity rate of around −1 dB in the 16–18 GHz range.
(4) With the prolongation of pre-polymerization time, the pore diameter decreases from 1.02 mm to 0.4 mm, exhibiting a typical normal distribution.
(5) The addition of CB increases the foam wall thickness, enhances its resistance to deformation, and thereby increases its strength and modulus.

## Figures and Tables

**Table 1:** Effect of CB content on foam compression performance.

CB Content (wt%)	Density (kg·m^−3^)	Foam Wall Thickness (mm)	Compression Strength (MPa)	Compression Modulus (MPa)
0	82.4 ± 2.1	0.014 ± 0.001	0.58 ± 0.02	16.0 ± 0.8
1	132.1 ± 3.2	0.021 ± 0.001	0.83 ± 0.05	26.1 ± 1.2
2	137.0 ± 3.4	0.023 ± 0.002	0.84 ± 0.06	31.7 ± 1.3
3	160.4 ± 4.5	0.032 ± 0.002	0.82 ± 0.04	28.5 ± 0.9

## Data Availability

Data are contained within the article.
